# IL-17A and Serum Amyloid A Are Elevated in a Cigarette Smoke Cessation Model Associated with the Persistence of Pigmented Macrophages, Neutrophils and Activated NK Cells

**DOI:** 10.1371/journal.pone.0113180

**Published:** 2014-11-18

**Authors:** Michelle J. Hansen, Sheau Pyng J. Chan, Shenna Y. Langenbach, Lovisa F. Dousha, Jessica E. Jones, Selcuk Yatmaz, Huei Jiunn Seow, Ross Vlahos, Gary P. Anderson, Steven Bozinovski

**Affiliations:** Lung Health Research Centre, Department of Pharmacology and Therapeutics, The University of Melbourne, Victoria, Australia; University of Rochester Medical Center, United States of America

## Abstract

While global success in cessation advocacy has seen smoking rates fall in many developed countries, persistent lung inflammation in ex-smokers is an increasingly important clinical problem whose mechanistic basis remains poorly understood. In this study, candidate effector mechanisms were assessed in mice exposed to cigarette smoke (CS) for 4 months following cessation from long term CS exposure. BALF neutrophils, CD4^+^ and CD8^+^ T cells and lung innate NK cells remained significantly elevated following smoking cessation. Analysis of neutrophil mobilization markers showed a transition from acute mediators (MIP-2α, KC and G-CSF) to sustained drivers of neutrophil and macrophage recruitment and activation (IL-17A and Serum Amyoid A (SAA)). Follicle-like lymphoid aggregates formed with CS exposure and persisted with cessation, where they were in close anatomical proximity to pigmented macrophages, whose number actually increased 3-fold following CS cessation. This was associated with the elastolytic protease, MMP-12 (macrophage metallo-elastase) which remained significantly elevated post-cessation. Both GM-CSF and CSF-1 were significantly increased in the CS cessation group relative to the control group. In conclusion, we show that smoking cessation mediates a transition to accumulation of pigmented macrophages, which may contribute to the expanded macrophage population observed in COPD. These macrophages together with IL-17A, SAA and innate NK cells are identified here as candidate persistence determinants and, we suggest, may represent specific targets for therapies directed towards the amelioration of chronic airway inflammation.

## Introduction

Chronic Obstructive Pulmonary Disease (COPD) is a debilitating lung condition that is characterized by chronic airway inflammation. COPD is now the third cause of death worldwide and kills more than 3.5 million people per year. About 85% of all COPD is caused by inhalation of irritants mostly cigarette smoke (active and passive), ambient air pollutants and poor indoor air quality caused by biomass cooking and heating fumes. Inflammation induced by these irritants contributes to key pathological processes in COPD including small airway narrowing, destruction of alveolar walls (emphysema) and mucous hypersecretion (reviewed in [1]). Innate immune cells including macrophages and neutrophils accumulate and are considered essential for disease progression [2], as are immune cells of the adaptive response including CD8^+^ T cells [3]. CD4^+^ T cells and B cells also aggregate and can organize into lymphoid follicles, the percentage of which increases with progression of COPD [2]. The close association of de novo lymphoid follicles with persistence and severity of COPD strongly suggests their contribution to deleterious autoimmunity in the airways although beneficial effects in terms of mounting a rapid immune response to respiratory pathogens have not been formally excluded. The combined activity of the these inflammatory cells is thought to drive the accelerated decline in lung function that is a hallmark of the disease.

Cigarette smoke (CS) cessation currently remains the single most effective strategy to reduce the accelerated decline in lung function attributable to COPD. At least in developed countries there is clear evidence that smoking rates have fallen, in part due to effective cessation strategies. However, cross-sectional and longitudinal studies have shown that in individuals with established disease, airway inflammation does not fully resolve with CS cessation [4], [5] and post cessation persistent lung disease is an increasingly important clinical problem. In particular, airway and sputum neutrophils persist and in some cases, increase with cessation [4]–[6]. Neutrophilic inflammation is particularly damaging in COPD due to a deficiency in efferocytosis (clearance of moribund cells) mediated by excessive oxidative stress [7], [8], which can lead to excessive degranulation of necrotic neutrophils. Activated neutrophils release neutrophil elastase and other serine proteases, which increases with the severity of COPD and these processes are intrinsically insensitive to inhaled glucocorticosteroids [9]. Neutrophil elastase degrades extracellular matrix components including elastin, collagens I–IV and fibrinogen and the degree of elastase localized to lung elastic fibers correlates with the degree of emphysema [10]. Neutrophil elastase can also promote mucin production [11] and activate TLR4-dependent production of IL-8 via epidermal growth factor receptor (EGFR) transactivation mechanisms [12].

Macrophages also accumulate in COPD airways and are positively associated with disease severity [2]. Importantly, depletion of macrophages protected against the development of emphysema in a chronic smoke exposure model; demonstrating a pathogenic role for this immune cell [13]. Furthermore, it is now recognized that macrophages acquire a distinct phenotype associated with the progressive induction of M2-related programs as a consequence of smoke exposure and COPD [14]. Macrophages can initiate neutrophilic inflammation as they are a major source of neutrophil chemokines. Several neutrophil chemokines such as IL-8 (CXCL8), KC (CXCL1) and MIP-2α (CXCL2) are implicated in COPD as they are elevated in CS exposure models [15] and during exacerbations [16]. In addition, Interleukin-17A (IL-17A) can promote neutrophil mobilization through its regulation of leukocyte growth factors and cytokines. Immunoreactive IL-17A^+^ cells increase in frequency in the submucosa of COPD patients [17] and IL-17A expression is elevated in CS exposure models, where mice lacking IL-17RA were protected from developing emphysema [18]. Serum Amyloid A (SAA) can also mobilize neutrophils into the airways, and SAA is elevated in COPD lung tissue [19] and is related to neutrophilic lung infiltration [20]. In this study, a CS cessation model was used to identify which molecular markers most closely relate to the persistence of innate immune responses. We identify IL-17A and SAA inflammatory cytokine networks in the persistence of inflammation following CS cessation and suggest that targeting these networks may be of therapeutic benefit in augmenting the benefit of smoking cessation in this disease group.

## Materials and Methods

### Animals

Specific pathogen-free male BALB/c mice obtained from the Animal Resource Centre (Perth, Australia) arrived at 6 weeks of age were housed at in sterile micro-isolator cages, and maintained on a 12∶12 h light/dark cycle. This study was carried out in strict accordance with the National Health and Medical Research Council (NHMRC) of Australia. All procedures were approved by the Animal Experimentation Ethics Committee of the University of Melbourne.

### Treatment

After a one week acclimatization period, mice were randomly divided into 4 groups (n = 14–18 per group) that were matched for body weight. Two groups of animals were exposed to cigarette smoke (CS) and two groups were sham exposed according to our published protocol [21], [22]. Briefly, animals underwent whole body exposure to the smoke of 1 filtered cigarette inside an 18 liter plastic chamber (Winfield Red, 16 mg or less of tar, 1.2 mg or less of nicotine and 15 mg or less of CO, Philip Morris) over 15 min with a 5 minute recovery interval and this was then repeated such that mice received 2 cigarettes over a 30 min period. Smoke was generated in 50-ml tidal volumes over 10 seconds by use of timed draw-back. The mean total suspended particulate (TSP) mass concentration in the chamber containing cigarette smoke generated from one cigarette, measured from 3 min 13 s to 15 min, was 419 mg/m^3^ as previously published [22]. This exposure protocol was repeated three times a day (8 am, 12 pm and 4 pm exposures) for 6 days a week and generates carboxyhemoglobin levels within the range observed in human smokers [22]. Sham animals were handled identically without cigarette smoke exposure. After 16 weeks of CS one group of mice was sacrificed, as described below. The remaining groups were then sacrificed after a period without CS of 4 and 12 weeks. Body weight was measured twice per week.

### Tissue Collection

The study protocol included 4 groups (n = 11–14 per group). Mice were weighed and given an anesthetic overdose (ketamine and xylazine, 180 and 32 mg/kg i.p., respectively) and allocated to the following experimental protocols. Cohort 1 (n = 8) were subjected to bronchoalveolar lavage (BAL). Briefly, lungs from each mouse were lavaged in situ with 0.4 ml PBS, followed by three 0.3 mL of PBS, with 1 ml of BAL fluid (BALF) recovered from each animal. Smoke exposure had no effect on the recovered volume as previously shown [22]. Whole lungs were perfused free of blood via right ventricular perfusion with 10 ml of saline, rapidly excised en bloc, blotted. The large left lobe was snap frozen in liquid nitrogen and stored at −80°C for QPCR analysis. The remaining lung tissue was retained and subjected to flow cytometry analysis as detailed in the flow cytometry methods section. Cohort 2 (n = 5–6) were subjected to histology as detailed in the histology methods section.

### Cellular Inflammatory Response

Bronchoalveolar lavage fluid (BALF) was collected as previously described [22]. Cytospins were prepared at 400 rpm for 10 min on a Cytospin 3 (Shandon, UK). Cytospin slides were stained with DiffQuik (Dade Baxter, Australia) and 500 cells per slide were by standard morphological criteria.

### Flow Cytometry

BALF cells were resuspended in FACS buffer (PBS 1% FCS). Lungs were perfused with ice-cold PBS to remove excess blood before single cell suspensions were obtained using collagenase. Briefly, whole lungs were digested with RPMI containing collagenase D (1 mg/mL) and DNase I (Roche, Mannheim, Germany) and cells were washed and recovered by centrifugation. Erythrocytes were lysed by incubation with RBC lysis buffer. To avoid non-specific binding of Abs to FcRγ, FACS Buffer containing anti-mouse CD16/32 mAb (Mouse BD Fc Block) (2.4G2, BD) was added to all primary stains. Cells were labeled with fluorophore-conjugated antibodies at pre-optimized dilutions to CD3-FITC, CD4-PE, CD8-PE, CD49b-PE (NK/NK T marker) and CD69-FITC (all from Becton Dickinson) for 1 h at 4°C and then washed twice in FACS buffer and resuspended in a final volume of 0.5 ml of FACS buffer. Data was acquired on a BD FACSCalibur flow cytometer (Becton Dickinson) and typically up to 10^5^ viable cell events were collected for analysis. A strict gating strategy was used to determine different immune cell populations as follows: single cell gate (FSC-H vs FSC-A), live cells (propidium iodide exclusion), granularity/size cell gate (FSC-A vs SSC-A) and specific surface marker gates. Flowjo software (version 7.2.4, Tree Star, OR) was used to generate plots for data analysis.

### Histology

Mouse lungs (n = 5–6 per group) were perfusion fixed *in* *situ* via a tracheal cannula with 10% neutral buffered formalin (NBF) at 25 cm H_2_O pressure. After 10 min, the trachea was ligated and the lungs were left *in* *situ* for 1 hr, then removed and immersed in 10% NBF for at least 24 hr and then embedded in paraffin. After paraffin embedding, 4 µm sections were prepared and stained with hematoxylin and eosin. The number of pigmented macrophages was counted by a treatment-blind observer at x200 magnification, with at least 8 fields captured per sample for analysis using ImageJ software. Assessment of the number of lymphoid follicles per mm^2^ of lung tissue was determined as previously published [23].

### Quantitative RT-PCR

Total RNA was isolated from lung tissue using an RNeasy kit (Qiagen, MD, USA) and was used as a template to generate first-strand cDNA synthesis using SuperScript III (Invitrogen, CA, USA). TaqMan low density arrays (Applied Biosystem, CA, USA) were used for determining gene expression of individual samples using an ABI 7900 HT Sequence Detection System (Applied Biosystems). Gene expression was quantified using 18S rRNA as an internal control as previously described [22].

### Statistical Analyses

Results are expressed as mean ± SE. All data were analyzed using two-way ANOVA and when statistical significance was achieved a *post hoc* Bonferroni test for multiple comparisons was used to compare between treatment groups. All statistical analyses were performed with GraphPad Prism for Windows (version 6.02). In all cases, probability values less than 0.05 (P<0.05) were considered statistically significant.

## Results

### Cessation of CS exposure restored body weight

**Figure 1:**
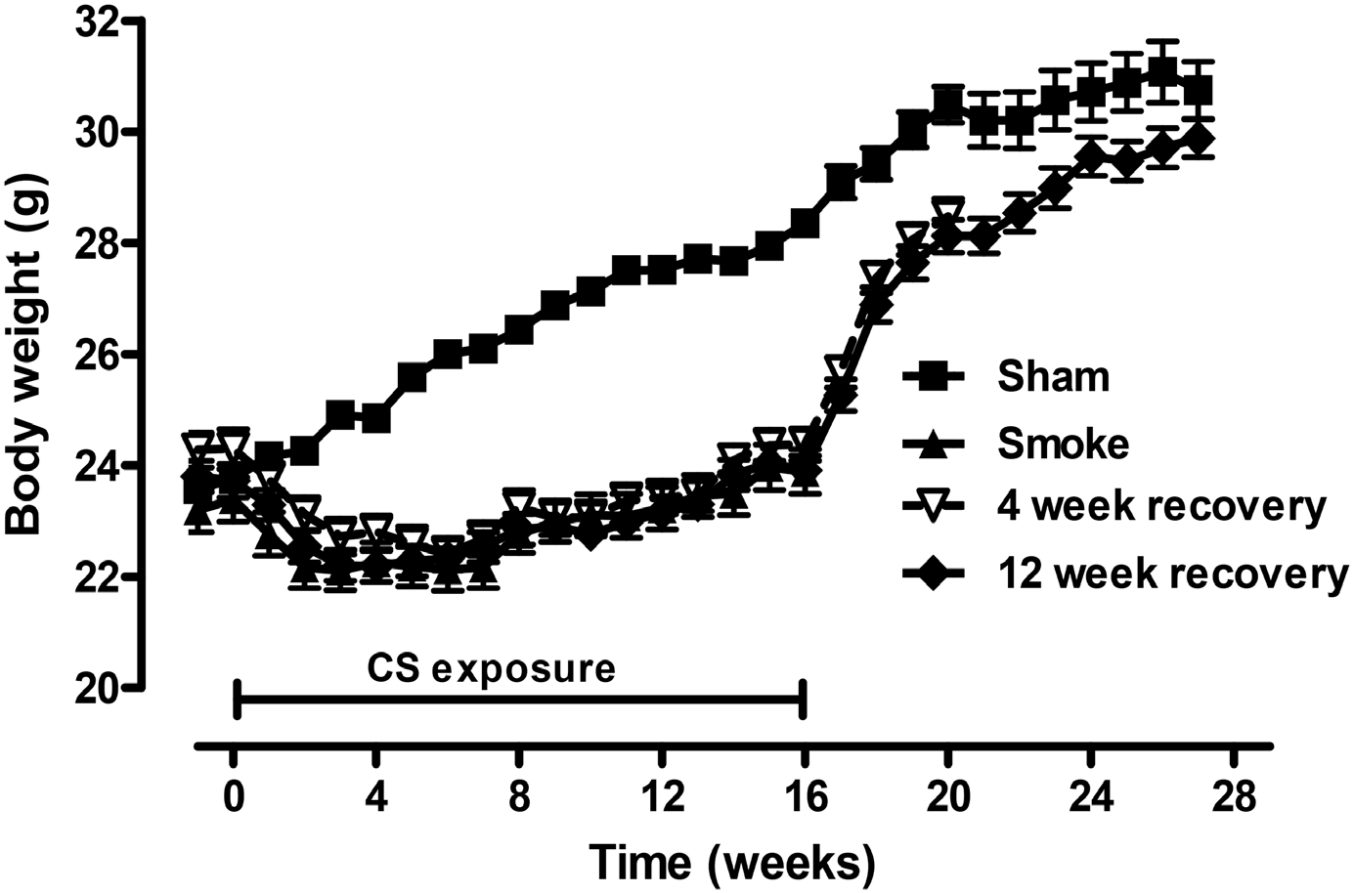
Smoke-induced weight loss was reversed 12 weeks after CS cessation. Male BALB/c mice were either exposed to 6 cigarettes/day, 6 days/week (▪) or sham handled (▴) for 16 weeks. After smoke exposure, groups of mice were then exposed to room air without cigarette smoke for either 4 weeks (∇) or 12 weeks (♦). For all groups body weight was determined weekly. Data are shown as mean ± SE for n = 14–18 per treatment group.

As previously reported [24], [25] mice exposed to CS failed to gain as much body weight as the Sham handled mice and were 15% lighter at the conclusion of the 16 week exposure period (P<0.05; Figure 1). After smoking cessation mice rapidly gained weight but remained significantly lighter by 6% after 4 weeks of recovery (P<0.05). By 12 weeks of recovery the body weight of mice previously exposed to CS was no different to the sham handled mice. Since the systemic effects of cigarette smoke resolve by 12 weeks, the cellular and molecular markers were characterized at this time point. In addition, following 16 weeks of smoke exposure, no significant increase in airspace enlargement was observed in BALB/c mice, which is consistent with previous studies that show an increase in mean linear intercept and destructive index in longer term chronic exposure models (i.e. 6 months) [26].

### Neutrophil and lymphocyte cell number remained elevated after 12 weeks of CS cessation

**Figure 2:**
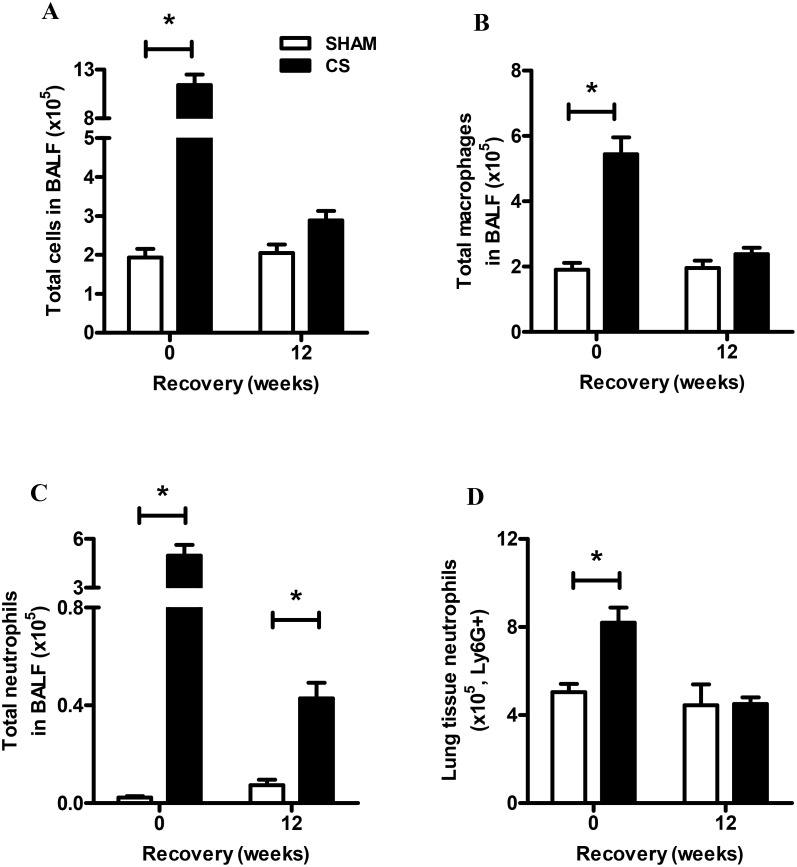
Effect of sub-chronic smoke exposure and 12 weeks of CS cessation on BALF cellularity and lung neutrophilia. Male BALB/c mice were either exposed to 6 cigarettes/day, 6 days/week (▪) or sham handled (□) for 16 weeks. After smoke exposure a group of mice was then exposed to room air without cigarette smoke for 12 weeks. Total cells (A), macrophages (B) and neutrophils (C) were determined in BALF. Data are shown as mean ± SE for n = 8–11 per treatment group. (D) Single cell suspension of the lungs was used to determine neutrophil numbers in the lung tissue by flow cytometry. Data were analysed by two-way ANOVA and when significance was achieved a *post hoc* Bonferroni test was performed. #P<0.05 significant *post hoc* effect of CS compared to sham animals at the same recovery time-point.

Mice exposed to CS for 16 weeks (6 cigarettes/day, 6 days/week) had a significant increase in total, macrophage and neutrophil number in BALF compared to sham mice (P<0.05, Figure 2A–C). Following 12 weeks of CS cessation BALF macrophage and total cell number decreased to sham levels. Peak neutrophil numbers in CS exposed mice declined by approximately 10-fold in the 12 weeks CS cessation group, however remained significantly elevated by 5-fold compared to Sham mice (P<0.05, Figure 2C). FACS analysis was used to determine the number of Ly6G+ neutrophils in the lung tissue, which showed that tissue neutrophils accumulated with CS exposure, resulting in a 1.6-fold increase above sham exposed mice (Figure 2D). Unlike neutrophil numbers in the BALF, tissue associated neutrophil numbers in the CS cessation group normalized to sham levels (Figure 2D).

**Figure 3:**
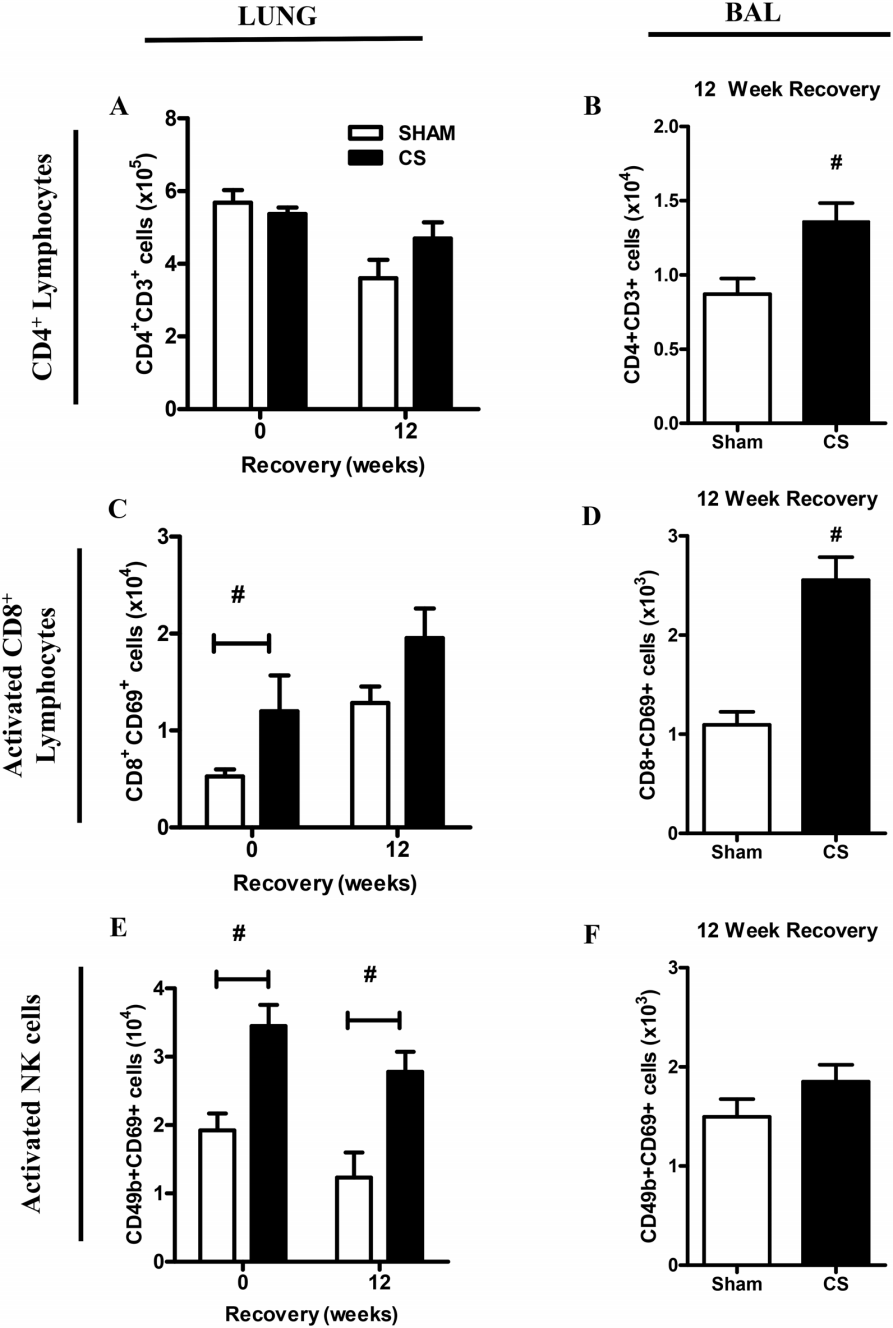
Effect of sub-chronic smoke exposure and 12 weeks of CS cessation on BALF and lung CD4^+^ and activated CD8^+^ lymphocytes. Male BALB/c mice were either exposed to 6 cigarettes/day, 6 days/week (▪) or sham handled (□) for 16 weeks. After smoke exposure a group of mice was then exposed to room air without cigarette smoke for 12 weeks. CD4^+^CD3^+^ lymphocyte number was determined in individual lung single cell suspensions (A) and BALF cells (B) using FACS analysis. Activated CD8^+^CD69^+^ lymphocyte number was determined in individual lung single cell suspensions (C) and BALF cells (D) using FACS analysis. In addition, activated NK cell numbers were quantified in individual lung single cell suspensions (E) and BALF cells (F). Data are shown as mean ± SE for n = 7–8 per treatment group. Data were analysed by two-way ANOVA and when significance was achieved a *post hoc* Bonferroni test was performed. #P<0.05 significant *post hoc* effect of CS compared to sham animals at the same recovery time-point.

FACS analysis was used to determine CD3^+^ CD4^+^ and activated CD69^+^CD8^+^ T cell number in BALF and lung tissue (Figure 3A–F). In lung tissue, CS had no effect on CD4^+^ T cell numbers and no change in frequency was observed in the cessation groups (Figure 3A). CD4^+^ T cells were also analyzed in the BALF compartment following 12 weeks CS recovery, demonstrating a 1.6-fold increase above Sham exposed mice (Figure 3B). Activated CD8^+^ T cells were also quantified by flow cytometry in the lung tissue, demonstrating a significant 2.3-fold increase in CS-exposed mice above Sham controls (Figure 3C). There was also a trend towards increased CD8^+^ T cell numbers in the CS cessation group (1.5-fold); however this failed to reach statistical significance. Analysis of BALF CD8^+^ T cells numbers demonstrated a 2.4-fold increase in the recovery group compared to the Sham controls (Figure 3D). In addition, activated NK cells were quantified in the lung and BAL compartment demonstrating a 2-fold increase in the lung tissue that was maintained in the CS cessation group (Figure 3E). In contrast there was no increase in activated NK cells in the BAL compartment (Figure 3F).

### Cigarette smoke exposure induced lymphoid aggregates and the prolonged elevation in pigmented macrophages

**Figure 4:**
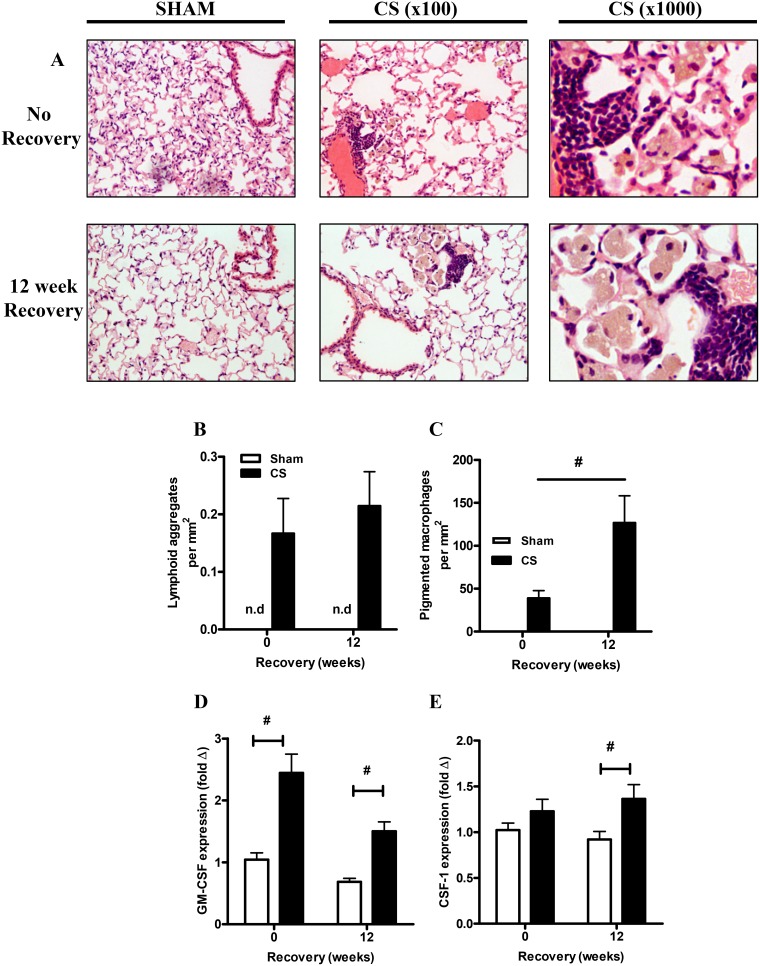
Sub-chronic smoke exposure resulted in the prolonged presence of pigmented macrophages. Representative histological staining of hematoxylin and eosin sections from sham and CS exposed mice and after 12 weeks of recovery (A). Magnification, x100 and x1000. The histological sections were scored for the number of lymphoid aggregates (B). The histological sections were scored for the presence of pigmented macrophages (C). Gene expression of the macrophage survival cytokines GM-CSF (D) and CSF-1 (E) was determined by Q-PCR, normalized to 18S rRNA and expressed as a fold change relative to the Sham no recovery group. Data are shown as mean ± SE for n = 7–8 per treatment group for QPCR and n = 4–6 for immunhistochemistry. Data were analysed by two-way ANOVA and when significance was achieved a *post hoc* Bonferroni test was performed. #P<0.05 significant *post hoc* effect.

Hematoxylin and eosin staining of lung sections revealed the presence of structures consistent with the formation of lymphoid aggregates, a hallmark of chronic inflammation in CS exposed mice, which persisted following 12 weeks of cessation (Figure 4A). Quantification of the number of tertiary lymphoid aggregates demonstrated that these structures appeared with 16 weeks of CS exposure and consistent with a recent study [23], persisted in the cessation group where numbers appeared to slightly increase over time (Figure 4B). The lymphoid aggregates were anatomically located in close proximity to pigmented macrophages. The accumulation of brown pigmented macrophages in the CS mice was quantified by a blind observer. CS exposure induced a significant increase in pigmented macrophages compared to sham mice (P<0.05, Figure 4C). Cessation resulted in a further 3-fold increase in the numbers of pigmented macrophages when compared to mice analyzed immediately after the 16 weeks of CS exposure (P<0.05, Figure 4C). Gene expression analysis of macrophage colony stimulating factors known to promote the survival and proliferation of leukocytes demonstrated that CS exposure caused a significant induction of GM-CSF mRNA compared to sham animals and this increase persisted following 12 weeks of cessation (P<0.05, Figure 4D). The mRNA expression of CSF-1 was also significantly induced after 12 weeks of CS cessation (P<0.05, Figure 4E).

### Differential effects of CS cessation on markers of alternative macrophage activation and neutrophil mobilization

**Figure 5:**
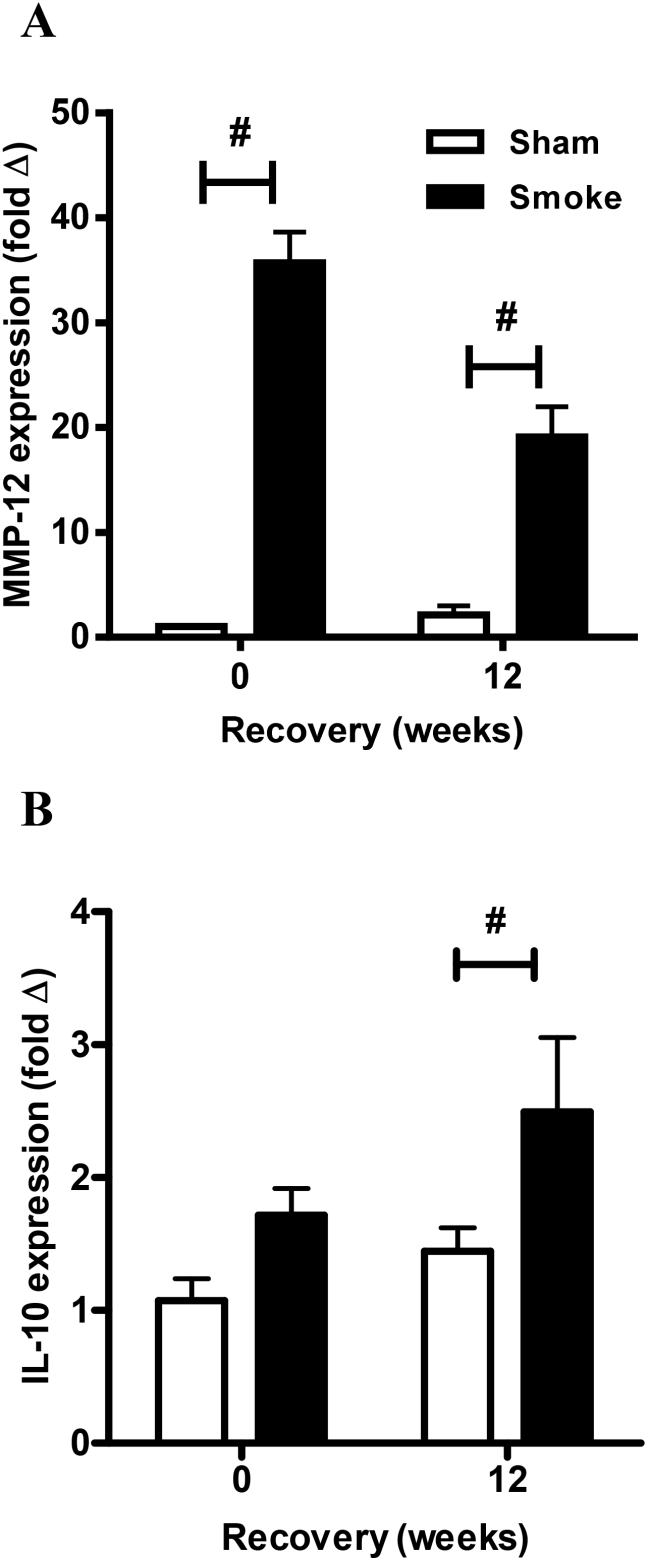
Effect of sub-chronic smoke exposure and 12 weeks of CS cessation on alternative macrophage marker mRNA expression in lung tissue. Male BALB/c mice were either exposed to 6 cigarettes/day, 6 days/week (▪) or sham handled (□) for 16 weeks. After smoke exposure a group of mice was then exposed to room air without cigarette smoke for 12 weeks. Gene expression of the alternative macrophage markers, MMP-12 (A) and IL-10 (B) was determined by Q-PCR, normalized to 18S rRNA and expressed as a fold change relative to the Sham 0 weeks recovery group. Data are shown as mean ± SE for n = 7–8 per treatment group. Data were analysed by two-way ANOVA and when significance was achieved a *post hoc* Bonferroni test was performed. #P<0.05 significant *post hoc* effect.

Expression of markers of alternative macrophage activation, MMP-12 and IL-10, was examined by QPCR of the lung tissue. CS caused a marked induction of MMP-12 gene expression (36-fold) and this remained significantly elevated by 19-fold after 12 weeks of cessation compared to sham animals (P<0.05, Figure 5A). IL-10 mRNA expression was also significantly elevated in the CS group after 12 weeks of smoking cessation compared to sham mice (P<0.05, Figure 5B).

**Figure 6:**
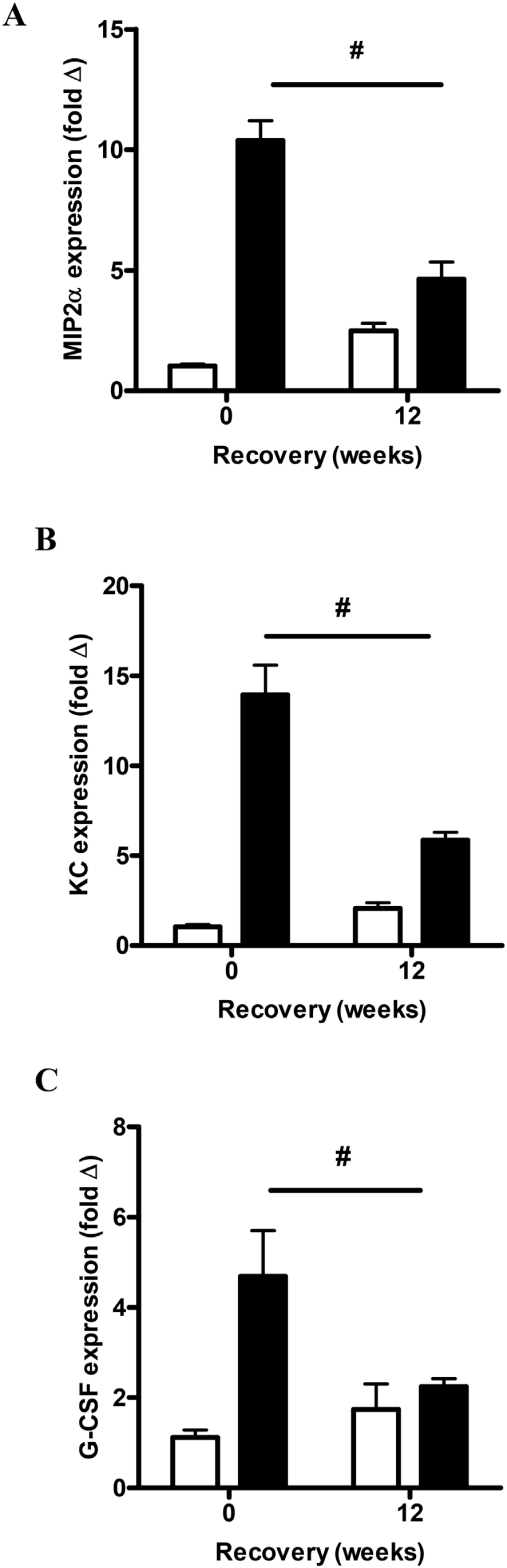
Effect of sub-chronic smoke exposure and 12 weeks of CS cessation on classic neutrophil mobilization mediators. Male BALB/c mice were either exposed to 6 cigarettes/day, 6 days/week (▪) or sham handled (□) for 16 weeks. After smoke exposure a group of mice was then exposed to room air without cigarette smoke for 12 weeks. Gene expression of MIP-2α (A), KC (B) and G-CSF (C) was determined by Q-PCR, normalized to 18S rRNA and expressed as a fold change relative to the Sham group. Data are shown as mean ± SE for n = 7–8 per treatment group. Data were analysed by two-way ANOVA and when significance was achieved a *post hoc* Bonferroni test was performed. #P<0.05 significant *post hoc* effect.

**Figure 7:**
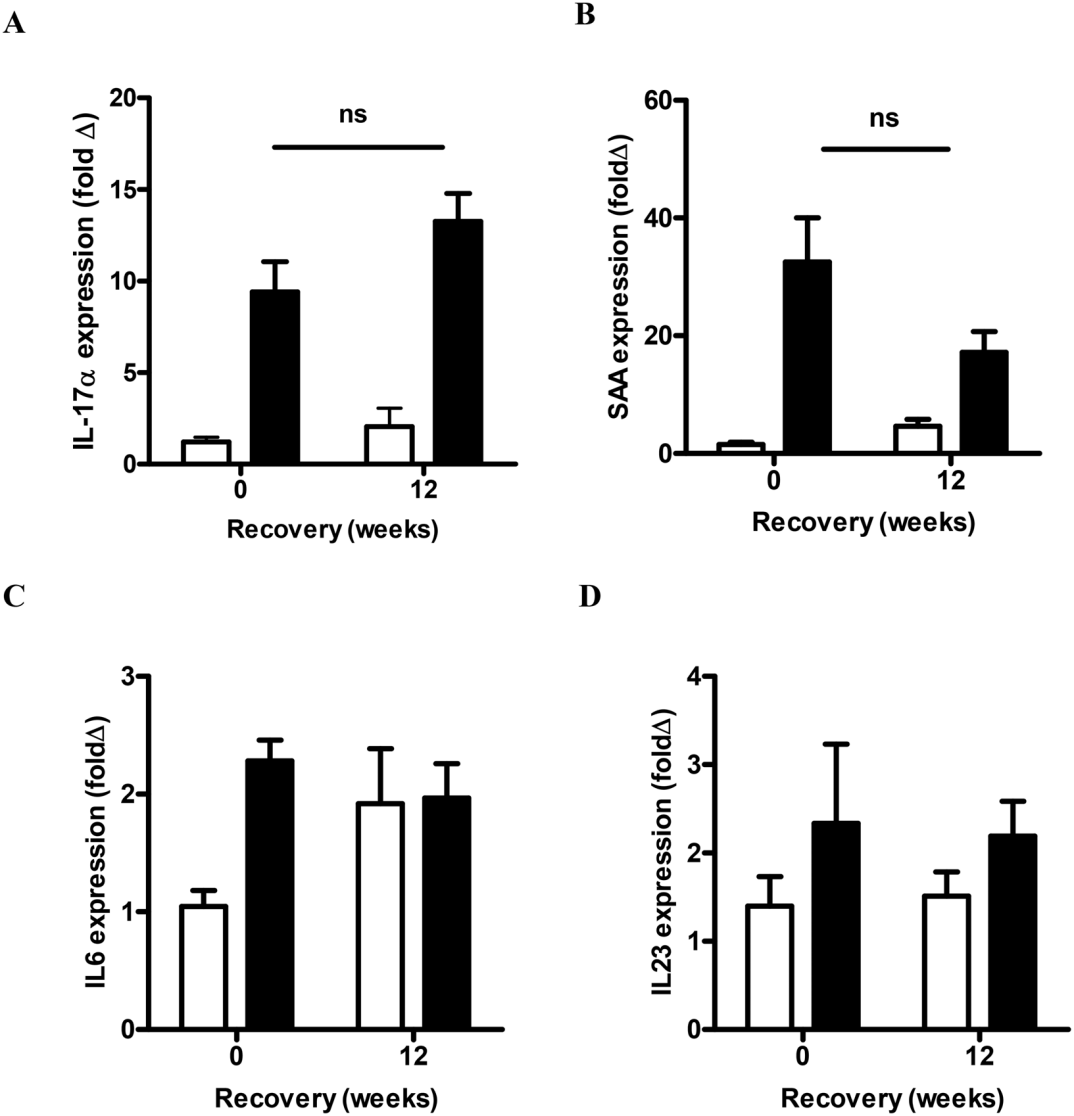
Effect of sub-chronic smoke exposure and 12 weeks of CS cessation on IL-17A and SAA expression. Male BALB/c mice were either exposed to 6 cigarettes/day, 6 days/week (▪) or sham handled (□) for 16 weeks. After smoke exposure a group of mice was then exposed to room air without cigarette smoke for 12 weeks. Gene expression of IL-17A (A), SAA (B), IL-6 (C) and IL23 (D) was determined by Q-PCR, normalized to 18S rRNA and expressed as a fold change relative to the Sham group. Data are shown as mean ± SE for n = 6–8 per treatment group. Data were analyzed by two-way ANOVA and when significance was achieved a *post hoc* Bonferroni test was performed.

The mRNA expression of neutrophil mobilization mediators was also examined. CS exposure significantly increased the mRNA expression of MIP-2α, KC and G-CSF compared to sham mice (P<0.05, Figure 6). 12 weeks of CS cessation resulted in a significant reduction in the mRNA expression of MIP-2α (2.3-fold), KC (2.3-fold) and G-CSF (2.1-fold) when compared to mice that were analyzed immediately following CS (P<0.05). Expression of the alternative neutrophil mobilizing mediators, IL17A and SAA, significantly increased by 9-fold and 33-fold above sham exposed mice respectively (P<0.05, Figure 7). IL-17A transcript levels did not decrease with CS cessation, where there was a 13-fold increase in the CS cessation group (Figure 7A). Although there was a trend towards reduced expression of SAA transcript in the CS cessation group (17-fold above sham), this was not significantly different to levels in CS exposed mice (Figure 7B). In addition, the well characterized T_H_17 polarising cytokines IL-6 (Figure 7C) and IL23 (Figure 7D) were measured by QPCR in the lung tissue. IL-6 levels were increased by 16 weeks of CS exposure; however there was no difference in IL-6 expression in the CS cessation arm. IL23 levels did not significantly increase with 16 weeks CS exposure.

## Discussion

COPD is a disease that displays a complex immunological profile associated with the engagement of innate and adaptive cellular processes in response to chronic CS exposure. Immune cells of both the innate and adaptive response persisted in our CS cessation model and this was previously associated with a modest reduction of alveolar enlargement and increased pulmonary compliance [27]. The innate response is particularly active in COPD, where macrophages and neutrophils accumulate in COPD airways [28], and neutrophilic inflammation fails to fully resolve in response to CS cessation [4], [5]. Our experimental model displayed a similar response to CS cessation where neutrophilic inflammation in the BAL compartment reduced with cessation but failed to fully resolve to control levels. In contrast, tissue neutrophils declined to control levels with CS cessation, which was consistent with the decline in G-CSF, a major hematopoietic growth factor required for mobilization and maturation of granulocyte precursors. Given the short-lived nature of blood derived neutrophils, the low level persistence of neutrophils in the BAL compartment is characteristic of an inflammatory response that has failed to fully resolve.

In addition, the adaptive response was also engaged, where increased CD4^+^ and CD8^+^ lymphocyte numbers in the BALF compartment remained elevated following CS cessation. Although total lymphocyte numbers in lung tissue were not significantly increased in the CS cessation group, there was an accumulation of lymphoid follicle-like structures in response to CS exposure that persisted in the cessation group. This is consistent with a recent report, where the persistence of lymphoid aggregates was associated with increased anti-nuclear autoantibody (ANA) production [23]. The role of these organized structures remain to be fully resolved, however therapeutic targeting of lymphoid follicle formation in mice chronically exposed to CS failed to suppress airway remodeling and alveolar enlargement [29]. There was also an increase in innate lymphoid NK cells in CS exposed mice, which persisted in the cessation group. This is consistent with the observed increase in NK cells in the induced sputum of COPD patients [30]. In a chronic CS challenge model, NK cells were shown to be more primed to release inflammatory mediators including IL-12 and IL-18 [31]. It has also been shown that the NK cell group 2D (NKG2D) ligand is increased in response to CS-exposure [32], which can sustain activation of cytotoxic T cells including NK cells.

CS models consistently show increased macrophage numbers in the BALF compartment (reviewed in [33]) and elevated macrophage numbers have been observed in other CS cessation models [27]. Here, we observed the persistence of pigmented macrophage populations that typically clustered together in regions adjacent to lymphoid aggregates. The presence of pigmented macrophages is thought to be related to the accumulation of CS products ingested by resident lung macrophages. To the best of our knowledge, this is the first study to quantify pigmented macrophages and demonstrate an increase with CS cessation. In conjunction with increased pigmented macrophage numbers, leukocyte colony stimulating factors, GM-CSF and CSF-1 transcript were significantly increased in the CS cessation group. Both CSFs are known to promote survival, proliferation and differentiation of myeloid lineages, and the findings presented here suggest that pigmented macrophages may proliferate in response to increased CSF expression. Whether these pigmented macrophages represent a distinct phenotype in COPD that contribute to disease pathobiology remains to be determined. There is however, growing evidence that macrophages do not conform to the classic M1/M2 dichotomy in COPD [14], [34]. In this study, IL-10 and MMP-12 expression were used as markers for differential macrophage polarization as previously reported [34], and increased expression suggest that alternative macrophage populations persist and contribute to chronic inflammation.

Previous global expression studies have shown that the majority of CS-inducible genes decline with cessation [35]. In our study, there was a focus on genes involved in neutrophil mobilization that are known to be upregulated in COPD. We have shown that IL-17A and SAA were not significantly reduced in the CS cessation group, in contrast to MIP-2α, KC and G-CSF that significantly declined with recovery. Our previous studies have demonstrated intense SAA immunoreactivity [19] and a positive correlation with neutrophilic airway inflammation [20] in the lungs of COPD patients. SAA is also a ligand for the GPCR termed ALX/FPR2, where SAA is a potent chemotactic factor that mediates phagocyte migration via this receptor [36]. SAA also promotes airway neutrophilic inflammation in a manner that is opposed by the eicosanoid, LipoxinA_4_ [19]. Lipoxins and resolvins are alternative lipid-based ALX/FPR2 ligands that can oppose the actions of SAA and actively promote the resolution of inflammation (reviewed in [37], [38]). Hence, the relative abundance of alternative ALX/FPR2 ligands may contribute to the impairment of resolution, where increased SAA may skew the balance towards a pro-inflammatory state.

SAA has also been shown to promote airway neutrophil recruitment via IL-17A dependent mechanisms [20]. There is also emerging evidence for an important role for IL-17A in COPD. IL-17A^+^ cells have been shown to be increased in the bronchial submucosa of chronic smokers and stable COPD subjects [17], [39]. Furthermore, genetic ablation of the IL-17R in experimental CS models protected the mice against the development of emphysema [18], hence identifying IL-17A as a major inflammatory cytokine that can drive pathological inflammation. Recent studies also demonstrate that neutrophilic inflammation induced by CS exposure is potently suppressed in mice deficient in IL-17A [40] and in response to neutralisation with a blocking antibody [41]. Furthermore, inhibition of IL-17A signaling in an experimental COPD model also suppressed accumulation of macrophages in response to CS exposure [18]. Our finding of persistent IL-17A expression in the CS cessation group is consistent with a recent study that identified an increase in the frequency of IL-17A expressing CD4^+^ (T_H_17) and CD8^+^ (T_C_17) T cells in CS exposed mice [42]. In our study, known T_H_17 cytokines were also quantified by QPCR and showed that IL-6, but not IL23 was significantly increased in response to CS exposure. This finding is consistent with our previous study that investigated T_H_17 cytokine expression in response to SAA stimulation, where IL-6 was predominately induced [20]. Although SAA levels were not significantly reduced with CS cessation, there was a trend towards reduced expression relative to the non-cessation group and IL-6 levels were not increased in the CS cessation group. This data suggests that SAA and IL-6 can be sufficient to initiate polarization and maturation of IL-17A expressing cellular populations in CS exposed lungs, however once established, IL-17A^+^ cells may be maintained in the mucosa independently of T_H_17 cytokines.

In addition to classic T_H_17 pathways, there is also emerging evidence for alternative innate cellular sources of IL-17A in inflammatory lung models. This may be particularly relevant to COPD as NOD. SCID mice deficient in B and T cells still develop airspace enlargement in response to chronic CS exposure, to suggest a more prominent role for innate immune responses [43]. Indeed, innate sources of IL-17A have been identified in inflammatory lung models including macrophages, neutrophils, NK cells and γδ T cells [20], [44] and the predominant source of IL-17A in COPD is yet to be defined. In conclusion, this study has investigated innate and adaptive responses following CS cessation and has identified the IL-17A and SAA innate cytokine networks as markers of persistent inflammatory responses. The targeting of the IL-17A axis may represent a novel therapeutic strategy to promote the resolution of inflammation following CS cessation.

## Supporting Information

Table S1 **Raw Data.**
(XLSX)
